# Transcriptional profile of breast muscle in heat stressed layers is similar to that of broiler chickens at control temperature

**DOI:** 10.1186/s12711-017-0346-x

**Published:** 2017-09-20

**Authors:** Imran Zahoor, Dirk-Jan de Koning, Paul M. Hocking

**Affiliations:** 1 Division of Genetics and Genomics, Roslin Institute and R(D)SVS, University of Edinburgh, Easter Bush, Midlothian, EH25 9RG UK; 2 Department of Animal Breeding and Genetics, University of Veterinary and Animal Sciences, Lahore, 54000 Pakistan; 3 Department of Animal Breeding and Genetics, Swedish University of Agricultural Sciences, 750 07 Uppsala, Sweden

## Abstract

**Background:**

In recent years, the commercial importance of changes in muscle function of broiler chickens and of the corresponding effects on meat quality has increased. Furthermore, broilers are more sensitive to heat stress during transport and at high ambient temperatures than smaller egg-laying chickens. We hypothesised that heat stress would amplify muscle damage and expression of genes that are involved in such changes and, thus, lead to the identification of pathways and networks associated with broiler muscle and meat quality traits. Broiler and layer chickens were exposed to control or high ambient temperatures to characterise differences in gene expression between the two genotypes and the two environments.

**Results:**

Whole-genome expression studies in breast muscles of broiler and layer chickens were conducted before and after heat stress; 2213 differentially-expressed genes were detected based on a significant (*P* < 0.05) genotype × treatment interaction. This gene set was analysed with the BioLayout Express^3D^ and Ingenuity Pathway Analysis software and relevant biological pathways and networks were identified. Genes involved in functions related to inflammatory reactions, cell death, oxidative stress and tissue damage were upregulated in control broilers compared with control and heat-stressed layers. Expression of these genes was further increased in heat-stressed broilers.

**Conclusions:**

Differences in gene expression between broiler and layer chickens under control and heat stress conditions suggest that damage of breast muscles in broilers at normal ambient temperatures is similar to that in heat-stressed layers and is amplified when broilers are exposed to heat stress. The patterns of gene expression of the two genotypes under heat stress were almost the polar opposite of each other, which is consistent with the conclusion that broiler chickens were not able to cope with heat stress by dissipating their body heat. The differentially expressed gene networks and pathways were consistent with the pathological changes that are observed in the breast muscle of heat-stressed broilers.

**Electronic supplementary material:**

The online version of this article (doi:10.1186/s12711-017-0346-x) contains supplementary material, which is available to authorized users.

## Background

Modern broiler chickens are characterised by relatively fast growth rate, greater muscle mass and better feed conversion ratio compared with layer and traditional chicken breeds [1, 2]. The carcasses of some broiler chickens show changes in the appearance of breast meat, such as a pale colour with reduced water holding capacity, or dark, firm and dry muscle with different functional properties [3]. More recently, white striping, which is characterised by white parallel striations in the direction of the muscle fibres and “wooden breast” muscles, have been reported [4, 5]. Elevated activity of creatine kinase and histopathological changes in affected muscles are suggestive of a degenerative myopathy [4, 6]. These changes have implications for meat quality and, potentially, have a significant economic cost. Several factors affect the proportion of affected carcasses, including different genetic background, growth rate, season, heat and transport stress, and abattoir practices [7–9].

Genetic variation in muscle and meat quality traits has been quantified [2, 10] but these traits usually involve measuring slaughtered sibs. Recent technological innovations have opened the way for genomic selection (GS) based on DNA markers (single nucleotide polymorphisms, SNPs) [11, 12]. Therefore, our objective was to identify genetic networks and pathways that might be useful for the detection of causal genetic factors that are involved in breast muscle and meat quality disorders of broiler chickens. It is also likely that the identified genetic factors would be helpful in updating the existing SNP chips to enable scientists to perform genomic selection for better muscle and meat quality in broilers.

Through the use of high-throughput microarray technology, it is possible to identify differentially-expressed genes as a result of a specific treatment [13]. In this study, we used microarray analysis to identify candidate genes that may contribute to differences in muscle damage between broilers and layers. Spontaneous and stress-induced myopathies in broiler skeletal muscles are exacerbated by heat stress [14, 15] and, thus, we compared gene expression profiles in the breast muscles of broiler and layer genotypes that were subjected to control or heat stress conditions. Our experimental strategy was based on the hypothesis that the expression of genes that are differentially expressed in broilers and layers under normal conditions is increased and therefore more easily detected after heat stress. However, it is often difficult to assign biological significance to the large number of genes that are detected in a microarray experiment. This problem can be solved when the differentially-expressed genes are organised via hierarchical clustering methods [16] and, for this purpose, we used BioLayout Express^3D^ [17, 18] and Ingenuity Pathway Analysis (IPA) (http://www.ingenuity.com/). In addition, we compared the results from these analyses with those obtained with the DAVID [19, 20] (https://david.ncifcrf.gov/) and Reactome [21, 22] (http://reactome.org/) software using more recent databases.

## Methods

### Animals and husbandry

We used 40 male broiler chicks of a male line (Ross 308, Aviagen, Newbridge, UK) from a commercial hatchery and 74 layer chicks (White Leghorn) from a line maintained at the Roslin Institute. For the first 2 weeks, birds were reared in groups of 20 individuals until the layers had been sexed by a DNA method [23]. At 2 weeks of age, the birds were distributed to eight pens by sex and genotype, with each pen containing 12 male layers and nine or ten broilers, in a completely randomised design. The birds were provided with feed (a commercial layer starter diet) and water ad libitum and the daily photoperiod was 16 h light and 8 h darkness.

The birds were subjected to experimental treatments over four days from 42 to 46 days of age. On each day, we randomly selected two pens for each breed and the birds were transferred into four controlled environment chambers. On each day, we randomly selected four chambers, i.e. two for the heat treatment (32 °C, 75% relative humidity or RH) and two as controls (21 °C, 50% RH). Each chamber contained two crates with two male broilers or two male layers, with pens and crates confounded. The crates were placed on a wooden pallet and the order of the pairs (crates) in each room was randomised. Sixty-four birds were used in the experiment.

About 30 min before the birds were transferred to the chambers, the relevant chamber was turned on, such that it could reach the required temperature and humidity before birds were placed into the chamber for the following 2 h. Birds were introduced in each chamber at intervals of 45 min to allow for sampling of the birds.

After completing the 2-h treatment, birds were removed from the crate and rectal temperatures were measured using a thermistor probe (Model 612-849; RS Components Ltd., Corby, Northants, UK). Then, they were euthanized by an intravenous injection of sodium pentobarbitone into the wing vein and two tissue samples of 100–120 mg were taken from the left pectoral muscle and snap frozen in liquid nitrogen for subsequent RNA extraction.

### RNA extraction and microarray experiment

Samples of breast muscle from male chickens were randomised prior to extraction of RNA using Trizol (Life Technologies, Paisley, UK) following the manufacturer’s recommended protocol. Briefly, the frozen tissue was homogenised in 1 ml of Trizol using the FastPrep^®^ system with Lysing matrix D (MP Biochemicals). The phases were separated by addition of 200 µl of 2-bromo-chloro-propane (Sigma Aldrich) and centrifuged for 15 min. A 500-µl sample of the clear upper aqueous layer was transferred to a fresh tube and 500 µl of isopropanol was added. The samples were centrifuged for 30 min to pellet the RNA, which was washed twice with 70% ethanol before air-drying. The RNA was resuspended in 100 µl of RNAse-free water prior to quantification and quality assessment. All RNA samples had a RNA integrity number (RIN) value higher than 8.0, as determined by the Agilent Bioanalyser RNA 6000 Nano Chip. Samples were diluted to 50 ng/μl with deionised and RNAse-free water. Aliquots of 20 μl from each sample were used for pooling the two samples from each crate to obtain eight replicates for each breed × treatment combination.

Microarray hybridisation was completed in the ArkGenomics laboratory at the Roslin Institute (http://genomics.ed.ac.uk **)**. Total RNA was prepared for hybridisation to the Affymetrix chicken GeneChip array using the Affymetrix IVT express kit according to the manufacturer’s protocol. The generated cRNA was hybridised overnight to the cartridge arrays according to Affymetrix’s protocols. The cartridges were washed and stained in the Affymetrix fluidic station using the hybridisation, wash and stain kit from Affymetrix. After staining, the arrays were scanned with the Affymetrix GeneChip system 3000 scanner. The resultant CEL files were reviewed using the Expression Console software from Affymetrix.

Thrity-two Affymetrix chicken array chips (38.5K; each GeneChip included 38,535 probes) were used in the microarray experiment. After scanning, the CEL files were analysed in four batches of eight slides to obtain expression values in GenStat (www.vsni.co.uk/software/genstat). Each batch contained slides from birds treated on the same day. The Robust Multichip Average (RMA) algorithm [24] was used to extract the gene expression data.

### Statistical analysis

The experiment was a 2 × 2 factorial design (breed × treatment), with day/chambers/crates as blocking factors. Standard analysis of variance methods was used to analyse body temperature and body weight using GenStat v13 (https://www.vsni.co.uk/software/genstat/). Transformation to natural logarithms was necessary to achieve normally distributed residuals of body weight.

For the analysis of differentially-expressed genes, we used a model with fixed effect terms for breed and treatment and their interaction. The normalised data were analysed by using Microarray One-Channel ANOVA in GenStat, with a model that included breed × treatment as treatment structure and the hierarchical structure of day/chamber/breed as blocking factor. Genes that showed a significant breed × treatment interaction (*P* < 0.05) were used for subsequent investigation because they were expected to be most relevant for genetic differences between broilers and layers in response to heat stress. Based on these ANOVA results, the false discovery rate (FDR) was calculated for three probability values (*P* < 0.05, <0.01 and <0.001) for the effects of treatment, breed, and their interaction. FDR was calculated using the Mixture Model of GenStat and the maximum number of iteration cycles was set to 300.

### Cluster analysis in BioLayout Express^3D^

Gene annotations were downloaded from the NetAffx analysis centre of Affymetrix (http://www.affymetrix.com/analysis/index.affx; downloaded 15 December 2016). Expression values for the selected subset of genes/probes were unlogged, entered into BioLayout Express^3D^ (BLE, http://www.biolayout.org/) and analysed using a Pearson correlation threshold of 0.80. Clusters were viewed in the Class Viewer, after running the Markov Clustering Algorithm (MCL). For cluster size, a minimum threshold of four genes/probes per cluster was selected to limit the size of the smallest clusters [25]. Selected clusters were identified on the basis of a clear difference in expression pattern of the genes between treatments (control vs. heat treatment) and breeds. For functional analyses, clusters were combined into ‘categories’ on the basis of similarity in mean expression pattern across breeds and treatments.

### Analysis of pathways and networks in IPA

The gene expression data for each of the six selected categories were combined into a single Excel sheet for analysis in Ingenuity Pathway Analysis (IPA, http://www.ingenuity.com/products/ipa) of the four breed × treatment combinations (broiler control, BC; broiler heat stress, BH; layer control, LC and layer heat stress, LH). The lists of genes for each category were analysed in IPA by using Fisher’s exact test to identify biological functions and pathways that were enriched in the dataset using the ‘Core Analysis’ function of the IPA program. Genes were mapped against the ‘Tissues and Cell Lines’ available in the Ingenuity Pathway Analysis Knowledge Base (IPAKB). Because information in the IPA originates mainly from mammals (human, mouse and rat), the submitted lists of genes were mapped against all available species and changes to avian terminology, e.g. neutrophil to heterophil, were made. For network generation, we set a threshold of 35 molecules per network and 25 networks per analysis. Both direct and indirect relationships of molecules were considered.

### Additional analyses of pathways and networks

To reconfirm the initial results, we repeated the analyses on pathways and networks with more recent databases. We used two software programs, i.e. DAVID (https://david.ncifcrf.gov/) and Reactome (http://reactome.org/), both accessed on 2nd July 2017. Further information is in Additional file 1: Table S1.

## Results

### Differentially-expressed genes

**Fig. 1:**
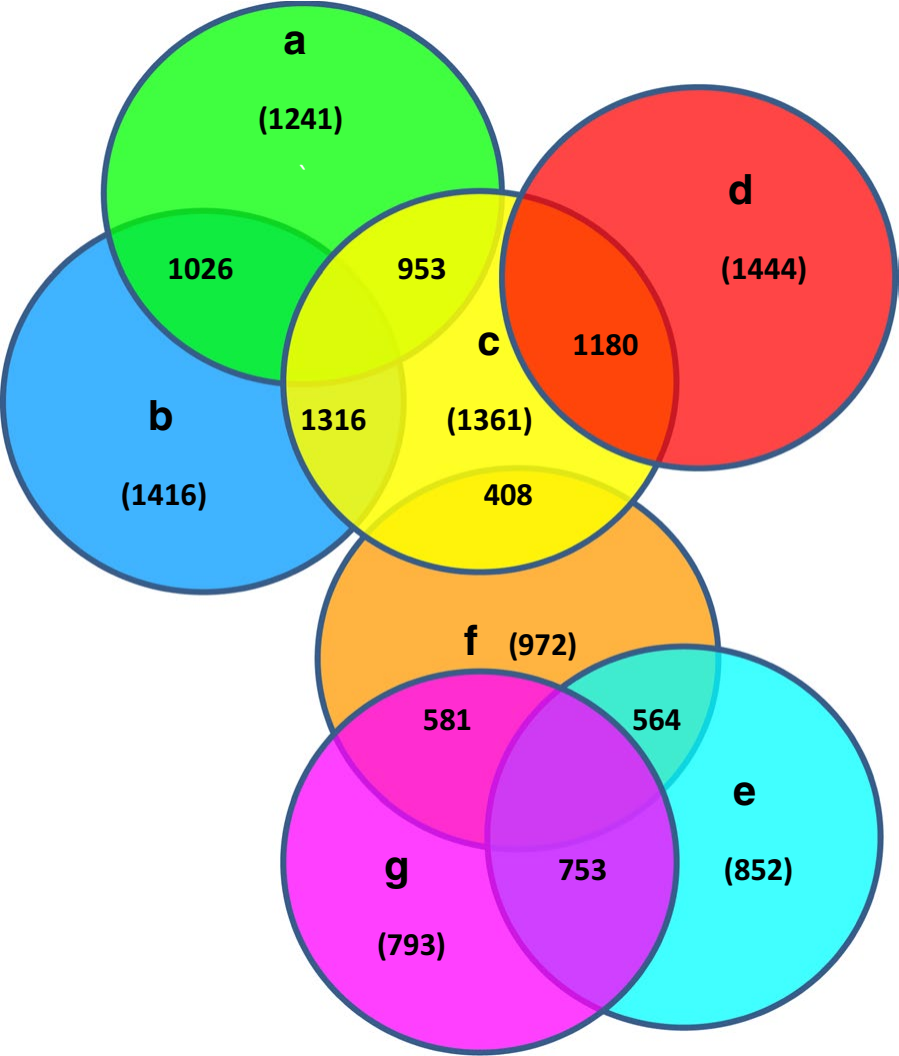
Differentially-regulated genes that overlap between two treatments. **a** Downregulated genes in broiler controls (BC) versus layer controls (LC). **b** Downregulated genes in heat-stressed layers (LH) compared with control layers (LC). **c** Upregulated genes in heat-stressed broilers (BH) compared with control broilers (BC). **d** Upregulated genes in heat-stressed broilers (BH) compared with heat-stressed layers (LH). **e** Downregulated genes in heat-stressed broilers (BH) compared with control broilers (BC). **f** Upregulated genes in broiler controls (BC) compared with layer controls (LC). **g** Upregulated genes in heat-stressed layers (LH) compared with layer controls (LC). The *number* in parentheses in *each circle* is the total number of differentially-expressed genes

The Affymetrix Genechips were filtered for expression levels higher than 1, which reduced the number of probes from 38,535 to 19,038. The results of the ANOVA for the filtered set of genes are in Table 1. The false discovery rate (FDR) for statistically significant genes (*P* < 0.05) was less than 31.5% for the treatment × breed interaction, 44% for treatment, and 3% for breed. A total of 2213 genes were differentially expressed among the four treatment comparisons. The numbers of differentially-regulated genes that overlapped between the two treatments are in Fig. 1. We found 1361 upregulated genes in the comparison between BH and BC, of which 1316 (97%) were shared with downregulated genes in the comparison between LH and LC. Similarly, we found 852 downregulated genes in the comparison between BH and BC, of which 753 (88%) were shared with upregulated genes in the comparison between LH and LC.

**Table 1:** Number of significant genes for treatment, breed, and breed × treatment interaction at different levels of significance

Significance (*P*<)	Treatment (heat-stress vs. control)	Breed (broiler vs. layer)	Breed × treatment interaction
0.001	107	5208	93
0.01	617	8182	635
0.05	1922	10,733	2213

### Categorisation of candidate genes on the basis of their biological functions

Based on their biological function, genes that were differentially expressed for the breed × treatment interaction were divided into 12 categories (Table 2). More than 43% (959) of the genes had no gene ontology (GO) term for a biological process or function. These genes fell in two major groups: 424 genes had no known function and 534 genes were not involved in a known biological process.

**Table 2:** Significant differentially-expressed genes for breed × treatment interaction (*P* < 0.05) grouped by function

Group	Biological functions	Number of genes
1	Transcripts with no known gene name	424
2	Genes with no GO terms for biological functions	534
3	Signal transduction	130
4	Stress-related response, inflammatory, angiogenesis, apoptotic, and proteolytic functions	334
5	Metabolic, and catabolic processes	190
6	Inter and intracellular transport of proteins, ions, and muscle contraction	162
7	Cellular proliferation, and organ development	142
8	Transcription and translation	138
9	Protein phosphorylation, dephosphorylation, modification, and folding	95
10	Signal transduction	92
11	DNA damage, repair, metabolism, and catabolic processes	60
12	Cytoskeleton organization and polymerization of filaments	42

### Comparisons of genes within and between breed and treatment significant for interaction

**Fig. 2:**
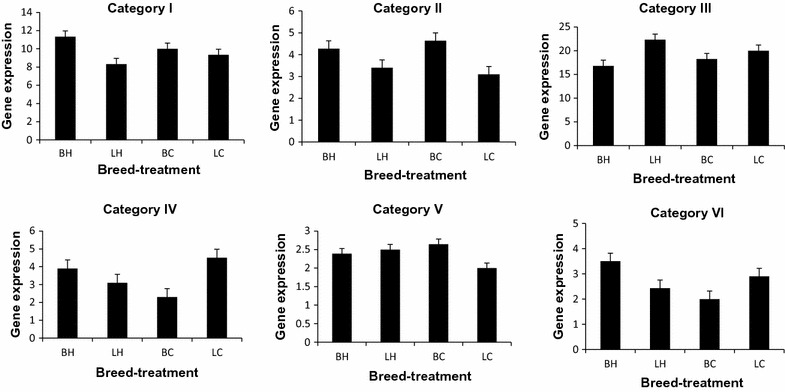
Mean expression levels of genes with significant breed × treatment interaction (*P* < 0.05) grouped into six categories (categories I–VI). Each *graph* has *four bars* and *each bar* represents one group. *BC* broiler control, *BH* heat-stressed broiler, *LC* layer control, *LH* heat-stressed layer

The selected genes were further divided into up- and downregulated patterns of gene expression for different comparisons within- and between-breed and treatment. Of the 54 clusters, 21 were selected for further analysis on the basis of their clear expression pattern, which included 509 genes that were grouped into six distinct categories (Table 3) according to the nature of their expression patterns corresponding to the (statistically significant) interactions of heat stress and genotype (Fig. 2). The expression values of the genes in category I were higher for broilers than for layers. Heat-stress resulted in a further increase in expression levels for broiler but a decrease for layers, compared to their respective controls. In the case of category II, the expression level of genes was higher for broilers than layers under control temperatures (as for category I). However, after heat-stress expression levels were lower in broilers compared with control broilers and conversely, higher in layers compared with LC. Expression values of category III genes were substantially higher for LC than BC whereas heat-stress resulted in further increases in gene expression in layers and decreases in broilers. Category IV genes were upregulated in BH compared with BC, whereas they were upregulated in LC compared with LH. Expression of category V genes was low in LC compared with all other groups and genes were upregulated in LH compared with LC, whereas they were downregulated in BH compared with BC. In the case of category VI genes, expression values for control layers were higher than for the respective broilers. After treatment, the expression of these genes increased in broilers but decreased in layers. Each of the six patterns of gene expression were analysed separately in IPA and significant (*P* < 0.05) pathways and networks were identified (see Additional file 1: Tables S2, S3, S4, S5, S6, S7). The set of genes which were filtered out by BioLayout Express (i.e. genes with a correlation coefficient of less than 0.80) was analysed in IPA separately, using the same procedure, to determine significant pathways and networks for this gene set, as shown in Additional file 1: Table S8.

**Table 3:** Numbers of genes, pathways and networks associated with different categories of genes based on function (see Fig. 2)

Category	Genes^a^	Pathways	Networks	Selected		
				Pathways	Networks	Functions
I	180	35	23	9	5	Stress response, cellular damage, connective tissue and muscle disorders
II	74	40	7	12	4	Cellular development, anti-apoptotic, anti-inflammatory and anti-stress functions
III	55	3	9	2	0	Anti-apoptotic, anti-oxidant, anti-inflammatory, energy production
IV	13	9	0	10	0	Stress, inflammatory, tissue damage, anti-oxidative, wound healing
V	7	9	4	5	0	Inflammation, immune functions, oxidative stress, phospholipid degradation
VI	16	7	4	3	0	Cell death, inflammatory and immune response, dellular development, haematopoiesis

^a^Genes mapped to corresponding identifiers

### Body weight and rectal temperature

Mean rectal temperatures for the control and heat-stressed conditions were 41.0 and 43.7 °C, respectively, in broilers, and 42.0 and 42.3 °C, respectively, in layers [standard error of difference (SED) 0.15 between breed and 0.14 between treatments]. The increase in rectal temperature in the heat-stressed birds was significantly larger in broilers (2.6 °C) than in layers (0.3 °C) which resulted in a significant breed × treatment interaction (*P* < 0.001). Average body weights (back-transformed) of broiler and layer males were 8.38 (4384 g) and 6.54 (693 g), respectively (SED 0.017, *P* < 0.001).

## Discussion

### Phenotypic responses validate experimental treatments

The large increase in rectal body temperature for broilers compared with layers is consistent with early reports in the literature [15, 26]. The results confirm the difficulty that broiler chickens have in coping with high ambient temperatures and other stressors, such as shackling, that may ultimately lead to detrimental consequences for both muscle function and meat quality [27, 28]. The results confirm that the heat treatment had the expected effect on the metabolism of broiler chickens and that the response in broilers was greater than in layers.

### Microarray analysis

The microarray results showed large differences between broilers and layers. Nevertheless, comparatively few significant genes (107, 617 and 1922 at *P* < 0.001, *P* < 0.01 and *P* < 0.05, respectively) were differentially-expressed in the comparison between treatments, which indicated that the differences in gene expression between heat-stressed and control birds were not as large as those between breeds. The number of upregulated genes in BH compared with BC (97%) that were shared with downregulated genes in the LH and LC comparison, and the number of downregulated genes in the former (BH vs. BC) compared with the latter (LH vs. LC) (88%), suggest that changes in gene expression in response to heat-stress are opposite in broilers compared to layers, which is consistent with the conclusion that broiler chickens do not manage heat stress appropriately. Furthermore, differential gene expression in breast muscles of BC and LH compared with LC, separately, involved a similar set of genes, which suggests that, in terms of gene expression, control broilers are similar to heat-stressed layers. We found that 1026 downregulated genes overlapped in the comparison of LH vs. LC (71%) and BC versus LC (83%) and likewise 753 genes were common/overlapped in the set of upregulated genes in the LH vs. LC (95%) comparison and in the list of downregulated genes in BH versus BC (88%) comparison. Taken together, these results are consistent with the physiological changes and muscle disorders that were reported for broiler chickens reared at conventional temperatures [1, 4].

The 2213 genes that were differentially-expressed for the breed × treatment interaction term were classified into categories according to their function and the biological processes in which they are involved. For 424 transcripts (19.2% of the total), we found no gene symbol and no gene name, which indicates that many genes involved in heat-stress induced responses in chicken skeletal muscle are not characterised to date. Similarly, the second largest group of genes, representing 13.1% of the significant genes, had no GO term for a biological function at the time the GO terms for this gene set were retrieved from the NetAffx Analysis Centre of Affymetrix (http://www.affymetrix.com/estore/analysis/index.affx, re-accessed 15th December 2016).

About 15% of the 2213 genes, which were significant (P < 0.05) for breed × treatment interaction, are directly involved in stress-related response, inflammatory, angiogenesis, apoptotic, and proteolytic functions, which is consistent with the physiological changes in broiler muscle caused by heat-stress [15, 29]. Similarly, 4% of the genes are involved in signal transduction and are associated with various biological processes, including oxidative stress, inflammation, muscle contraction, glycogen metabolism, and the concentrations of intracellular ions [30–34], and 7.3% are involved in inter and intracellular transport of proteins associated with muscle contraction and muscle damage-related functions [35–37]. Other smaller categories of genes are involved in cellular proliferation, development and DNA damage repair.

Stress is known to accelerate metabolic rate, mainly through carbohydrate metabolism to produce larger amounts of energy and facilitate “fight or flight” responses [38–40]; about 6% of all 2213 genes were involved in metabolic and catabolic functions. The cytoskeleton is required for cell shape and motility and is involved in cell division [41, 42]. It has been suggested that the genes in Group 11 (Table 2) have a role in the movement and division of leukocytes, such as heterophils and macrophages, as secondary mediators of the genes in Groups 3, 4 and 5 to shape the stress and inflammatory response to heat stress. Of all the significant genes for breed × treatment interaction, 49 encode proteins located in the mitochondria and about 200 affect the cell membrane directly. These results suggest that damage to mitochondria and cell membrane are potentially important components of heat-stress induced pathogenesis in chicken breast muscles.

Taken together, these results suggest a picture of stress responses, inflammation, oxidative stress, and tissue damage, which is consistent with histological and physiological changes in broiler breast muscle [1]. Confirmatory evidence was also reported in a recent IPA analysis of differentially-expressed genes in “wooden breast” and control broiler muscles [43].

### IPA analysis

Heat stress in broilers led to further increases in the expression of category I genes of the α-adrenergic signalling network (see Additional file 1: Table S2), which are involved in glycogenolysis under stressful conditions to provide energy for muscle contraction. However, stress hormones are also known to alter the activities of immune cells and lead to the production of various pro-inflammatory cytokines and chemokines [44, 45]. In agreement with these findings, genes of several chemokine pathways were also present in this category, which are involved in cytokine signalling, tissue damage and related functions (see Additional file 1: Table S2). Upregulation of these pathways in control broilers indicates that breast muscles in broilers at conventional ambient temperatures show physiological and functional changes that are further exacerbated by exposure to heat stress. However, the upregulated vascular endothelial growth factor (*VEGF*) signalling pathway is also a significant mediator of hypoxia-induced angiogenesis and is usually upregulated in hypoxia-like situations. Upregulation of this pathway in control broilers compared with layers suggests that broiler muscle cells were under hypoxic-stress even under control conditions. The reason for this may lie in the larger size of muscle fibres in broilers and an inadequate capillary supply, which are, in turn, considered to induce metabolic stress due to the larger diffusion distances for nutrients, metabolites and waste products [1]. This is consistent with reports that thermal stress leads to oxidative stress and muscle damage, as indicated by higher plasma creatine kinase activity [26, 46–48]. Upregulation of the *nuclear factor erythroid 2*-*related factor 2* (*NRF2*)-mediated oxidative stress response pathway may be a protective measure to minimise the damaging effects of heat stress on anti-oxidant functions [49–51].

Expression of category II genes was highest in BC and decreased after heat stress. Upregulation of protein synthesis and angiogenic pathways in BC is logical, in the sense, that broilers have substantially higher growth rates and larger body mass than layers [52, 53]. Exposure to heat stress resulted in downregulation of these pathways in broilers, which is consistent with the negative effects of heat stress on growth-related traits [54]. Conversely, inflammatory and anti-inflammatory pathways were upregulated in layers after heat stress, possibly as a mechanism to protect the body from tissue damage. However, these results are in agreement with the physiological data (body temperature) from the current study that show that the increase in body temperature was much smaller in layers than in broilers. Consistent with this, Sandercock et al. [14] reported that the effects of heat stress on body temperature and plasma creatine kinase activity were much smaller in layers than in broilers. Similarly, the extent of heat stress induced oxidative stress in skeletal muscles was much smaller in layers than in broilers [55].

Hypoxia is known to decrease the efficiency of oxidative phosphorylation [56] and, thus, the downregulation of this pathway in broilers (Additional file 1: Table S4) could be due to hypoxia-like conditions in skeletal muscles. In contrast to our finding, Toyomizu et al. [57] reported that oxidative phosphorylation in skeletal muscles was much more efficient in broilers than in layers at 14–28 days of age when body weights were about 1.0 and 0.2 kg for broilers and layers, respectively. This greater efficiency of oxidative phosphorylation in broilers at that age is a logical outcome of selection for rapid growth. In the present study, broilers at 6 weeks of age were over 3.5 kg heavier than 28-day layers and the occurrence of an hypoxia-like situation in their muscles is consistent with a higher muscle to capillary ratio and larger diffusion distances for nutrients and metabolic wastes [1]. Consistent with this conclusion, some angiogenic pathways in category I that are involved in hypoxia-induced angiogenesis, such as the *VEGF* signalling pathway, were upregulated in broilers.

Category VI genes, such as *Janus kinase 1* (*JAK1)*, *Janus kinase 2* (*JAK2)* and *tyrosine kinase 2* (*TYK2*) were up-regulated in BH compared with BC and have a role in wound healing and tissue regeneration [58], in agreement with categories I and IV genes, which indicates that muscle damage is much more important in heat-stressed broilers than BC, LC and LH. However, these pathways were downregulated in LH compared with LC, possibly because upregulation of survival-related pathways reduced damaging effects in LH [59].

## Conclusions

The experimental paradigm of combining genetic and environmental differences was successful in identifying a limited number of pathways and networks that underlie muscle function and meat quality. Our findings provide new insights into the genetics and pathogenesis of muscle damage induced by heat stress through the identification of previously unknown pathways and networks. Importantly, our study also showed that the gene expression pattern for breast muscle of broiler chickens that were raised under a conventional (control) temperature was similar to that of heat-stressed layers and that the expression of these genes was further enhanced in heat-stressed broilers. These results provide a resource for the identification of candidate genes for muscle function and meat quality, which we will use in an accompanying paper to determine statistically significant associations of SNPs with muscle and meat quality traits in chicken.

## Additional file

**Additional file 1: Table S1.** Comparison of pathways analyses of microarray (gene expression) data on IPA, DAVID, and REACTOME. **Table S2**. Pathways selected from category I cluster analysis. **Table S3**. Pathways selected from category II cluster analysis. **Table S4**. Pathways selected from category III cluster analysis. **Table S5**. Pathways selected from category IV cluster analysis. **Table S6**. Pathways selected from category V cluster analysis. **Table S7**. Pathways selected from category VI cluster analysis. **Table S8**. Selected pathways from IPA analysis of Biolayout filtered genes.
