# Supplementary material for: Transcriptional profile of breast muscle in heat stressed layers is similar to that of broiler chickens at control temperature
Source: Genet Sel Evol. 2017 Sep 20;49:69. doi: 10.1186/s12711-017-0346-x (PMC5607596; doi:10.1186/s12711-017-0346-x)
Supplement: Supplementary file 1 — Additional file 1: Table S1. Comparison of pathways analyses of microarray (gene expression) data on IPA, DAVID, and REACTOME. Table S2. Pathways selected from category I cluster analysis. Table S3. Pathways selected from category II cluster analysis. Table S4. Pathways selected from category III cluster analysis. Table S5. Pathways selected from category IV cluster analysis. Table S6. Pathways selected from category V cluster analysis. Table S7. Pathways selected from category VI cluster analysis. Table S8. Selected pathways from IPA analysis of Biolayout filtered genes. [file 12711_2017_346_MOESM1_ESM.docx]

**Table S1 Comparison of pathways analyses of microarray (gene expression) data on IPA, DAVID, and REACTOME**

| **Genes** | **Features** | **Pathway analysis tools** | | |
| --- | --- | --- | --- | --- |
|  |  | **IPA**  **(analyzed in 2010-2011)** | **DAVID^1^**  **(analyzed on 02-07-2017)** | **REACTOME^2^(pluggedInCytoscape)**  **(analyzed on 02-07-2017)** |
| Category 1 | Total genes | 286 | 286 | 286 |
|  | Genes mapped | 194 | 241 | 231 |
|  | Genes unmapped | 92 | 45 | 55 |
|  | Pathways at *P*<0.05 | 35 |  | 61 |
|  | Total pathways | 207 | 77 | 84 |
|  | *P*-value^3^ | Yes provided | Not provided | Yes provided |
|  | Ratio^4^ | Yes provided | Not provided | Yes provided |
|  | Total networks | 23 | --- | 1 |
| Category 2 | Total genes | 93 | 93 | 93 |
|  | Genes mapped | 75 | 85 | Not analyzed by Reactome |
|  | Genes unmapped | 18 | 8 |  |
|  | Pathways at *P*<0.05 | 40 | No P-values are available |  |
|  | Total pathways | 195 | 27 |  |
|  | P-value^3^ | Yes provided | Not provided |  |
|  | Ratio^4^ | Yes provided | Not provided |  |
|  | Total networks | 7 | --- |  |
| Category 3 | Total genes | 63 | 79 | 79 |
|  | Genes mapped | 57 | 64 | Not analyzed by Reactome |
|  | Genes unmapped | 9 | 15 |  |
|  | Pathways at *P*<0.05 | 3 | --- |  |
|  | Total pathways | 61 | 21 |  |
|  | P-value^3^ | Yes provided | Not provided |  |
|  | Ratio^4^ | Yes provided | Not provided |  |
|  | Total networks | 9 | ---- |  |
| Category 4 | Total genes | 23 | 23 | 23 |
|  | Genes mapped | 13 | 23 | Not analyzed by Reactome |
|  | Genes unmapped | 10 | 6 |  |
|  | Pathways at *P*<0.05 | 106 | 10 |  |
|  | Total pathways | 10 | Not provided |  |
|  | P-value^3^ | Yes provided | Not provided |  |
|  | Ratio^4^ | Yes provided | Not provided |  |
|  | Network yielded |  |  |  |
| Category 5 | Total genes | 10 | 10 | 10 |
|  | Genes mapped | 8 | 8 | Not analyzed by Reactome |
|  | Genes unmapped | 2 | 2 |  |
|  | Pathways at *P*<0.05 | 106 | 3 |  |
|  | Total pathways | 9 | --- |  |
|  | P-value^3^ | Yes provided | Not provided |  |
|  | Ratio^4^ | Yes provided | Not provided |  |
|  | Total networks | 4 | Not provided |  |
| Category 6 | Total genes | 18 | 18 | 18 |
|  | Genes mapped | 16 | 17 | Not analyzed by Reactome |
|  | Genes unmapped | 2 | 1 |  |
|  | Pathways at *P*<0.05 | 20 | 5 |  |
|  | Total pathways | 7 | --- |  |
|  | P-value^3^ | Yes provided | Not provided |  |
|  | Ratio^4^ | Yes provided | Not provided |  |
|  | Total networks | 4 | Not provided |  |
| Biolayout filtered | Total genes | 1066 | 1066 | 1066 |
|  | Genes mapped | 644 | 840 | 771 |
|  | Genes unmapped | 422 | 226 | 295 |
|  | Pathways at *P*<0.05 | See note 5 | 250 | 81 |
|  | Total pathways | 32 | --- | 60 |
|  | P-value^3^ | Yes provided | Not provided | Provided |
|  | Ratio^4^ | Yes provided | Not provided | Provided |
|  | Total networks | 25 | --- | 2 |

1. DAVID: No *P*-value and ratios are provided by DAVID. Moreover, unlike IPA, networks are not provided by DAVID.
2. Reactome: The software takes the Gene Symbols in its input file not the Probe Ids like IPA and DAVID. Hence, the Probe Ids, which do not have an official Gene Symbol, cannot be analyzed by Reactome.

The data for categories 2, 3, 4, 5, and 6 were not analysed by Reactome due to the small size of the gene set in these categories.

1. *P*-value: Shows the strength of the association between a specific pathway and the uploaded dataset. Small *P* values suggest that the probability of a spurious association between the data and this pathway is unlikely (<http://ingenuity.force.com/ipa/>).
2. Ratio: The number of genes in a given pathway divided by the total number of genes that make up that pathway
3. The complete list of pathways yielded by IPA was not downloaded because for this category even the number of significant pathways was reasonably large enough.

Table S2 Pathways selected from category I cluster analysis

| **No.** | **Pathway title** | ***P*-value** | **Ratio** | **Functions and disease** |
| --- | --- | --- | --- | --- |
| 1 | Fcγ receptor-mediated phagocytosis in macrophages and monocytes | 0.000022 | 0.078 | Inflammatory response [[60](#_ENREF_60)]; cellular immune response [[61](#_ENREF_61)]; phagocytosis [[62](#_ENREF_62)] |
| 2 | fMLP signalling in neutrophils | 0.000057 | 0.062 | Involved in inflammatory diseases [[63](#_ENREF_63)]; causes degranulation and production of tissue-destructive oxygen-derived free radicals [[64](#_ENREF_64)]. |
| 3 | CCR5 signalling in macrophages | 0.0067 | 0.043 | Inflammation; calcium influx; activation of PYK2; |
| 4 | CD28 signalling in T Helper cells | 0.0107 | 0.038 | Cellular immune response [[65](#_ENREF_65)] |
| 5 | CCR3 signalling in eosinophils | 0.0186 | 0.039 | Inflammation, tissue damage by release of reactive oxygen species (ROS) and toxic granule proteins [[66](#_ENREF_66)]; connective tissue diseases of unknown origin [[67](#_ENREF_67)]. |
| 6 | α-adrenergic signalling | 0.0228 | 0.038 | Stress response, glycogenolysis; rise in intracellular Ca^2+^ [[68](#_ENREF_68)] |
| 7 | NRF2-mediated oxidative stress response | 0.0282 | 0.031 | Transactivates detoxifying and antioxidant enzymes, such as heme oxygenase and superoxide dismutase [[69](#_ENREF_69)] |
| 8 | VEGF signalling | 0.0326 | 0.04 | Mediator of hypoxia-induced angiogenesis, proliferation of endothelial cells [[70](#_ENREF_70)] |
| 9 | Role of MAPK signalling in the pathogenesis of influenza | 0.0374 | 0.046 | Apoptosis, production of prostaglandin E2 [[71](#_ENREF_71)] |
| 10 | Chemokine signalling | 0.0472 | 0.041 | Inflammatory response [[72](#_ENREF_72)] |

^1^*P*-value: Shows the strength of the association between a specific pathway and the uploaded dataset. Small *P* values suggest that the probability of a spurious association between the data and this pathway is unlikely (<http://ingenuity.force.com/ipa/>).

^2^Ratio: The number of genes in a given pathway divided by total number of genes that make up that pathway (http://ingenuity.force.com/ipa/).

Table S3 Pathways selected from category II cluster analysis

| **No.** | **Pathway title** | ***P*-value^1^** | **Ratio^2^** | **Functions and disease** |
| --- | --- | --- | --- | --- |
| 1 | Ceramide signalling | 0.0007 | 0.046 | Apoptosis; cell cycle regulation; inflammatory response [[73](#_ENREF_73), [74](#_ENREF_74)] |
| 2 | Regulation of eIF4 and p70S6K signalling | 0.0024 | 0.03 | Cellular growth and development; protein synthesis [[75](#_ENREF_75)] |
| 3 | p70S6K signalling | 0.0036 | 0.031 | Cellular development; protein synthesis [[76](#_ENREF_76)] |
| 4 | EIF2 signalling | 0.0097 | 0.03 | Protein synthesis [[77](#_ENREF_77)] |
| 5 | HMGB1 signalling | 0.0119 | 0.03 | Inflammatory response; cellular immune response; cytokine signalling; proliferation of vessel-associated stem cells; muscle tissue regeneration [[78-80](#_ENREF_78)] |
| 6 | Sphingosine-1-phosphate signalling | 0.0172 | 0.025 | Inhibition of caspases mediated apoptosis; angiogenesis; development and functions of cardiovascular system [[81-84](#_ENREF_81)]. |
| 7 | Role of PI3K/AKT signalling | 0.0172 | 0.021 | Involve in protein synthesis and angiogenesis and inhibition of apoptosis [[71](#_ENREF_71), [85](#_ENREF_85)]. |
| 8 | Relaxin signalling | 0.0258 | 0.019 | Inhibition of histamine release [[86](#_ENREF_86)]; connective tissue remodelling; stimulation of angiogenesis; modulation of blood pressure [[87](#_ENREF_87)] |
| 9 | CNTF signalling | 0.029 | 0.036 | Enhances cell survival; prevents degeneration of skeletal muscle cells [[88](#_ENREF_88), [89](#_ENREF_89)] |
| 10 | Role of MAPK signalling in the pathogenesis of influenza | 0.0333 | 0.031 | Cell death; inflammatory response; production of prostaglandin E2 [[71](#_ENREF_71)]. |
| 11 | Chemokine signalling | 0.0395 | 0.027 | Inflammatory response [[90](#_ENREF_90), [91](#_ENREF_91)] |
| 12 | Production of nitric oxide and reactive oxygen species in macrophages | 0.0447 | 0.016 | Cellular immune response; production of NO and ROS in macrophages; free radical scavenging [[92](#_ENREF_92), [93](#_ENREF_93)] |

^1^*P*-value: Shows the strength of the association between a specific pathway and the uploaded dataset. Small *P* values suggest that the probability of a spurious association between the data and this pathway is unlikely (<http://ingenuity.force.com/ipa/>).

^2^Ratio: The number of genes in a given pathway divided by total number of genes that make up that pathway (http://ingenuity.force.com/ipa/).

Table S4 Pathways selected from category III cluster analysis

| **No.** | **Pathway title** | ***P*-value^1^** | **Ratio^2^** | **Functions and diseases** |
| --- | --- | --- | --- | --- |
| 1 | Estrogen receptor signalling | 0.0378 | 0.015 | Anti-oxidant functions, anti-apoptotic activities, anti-stress activity [[94-96](#_ENREF_94)] |
| 2 | Oxidative phosphorylation | 0.0468 | 0.013 | Energy production [[57](#_ENREF_57), [97](#_ENREF_97)] |

^1^*P*-value: Shows the strength of the association between a specific pathway and the uploaded dataset. Small *P* values suggest that the probability of a spurious association between the data and this pathway is unlikely (<http://ingenuity.force.com/ipa/>).

^2^Ratio: The number of genes in a given pathway divided by total number of genes that make up that pathway (http://ingenuity.force.com/ipa/).

Table S5 Pathways selected from category IV cluster analysis

| **No.** | **Pathway title** | ***P*-value^1^** | **Ratio^2^** | **Functions and diseases** |
| --- | --- | --- | --- | --- |
| 1 | Melatonin signalling | 0.000498 | 0.026 | Scavenges oxygen-centred free radicals, especially the highly toxic hydroxyl radical [[98](#_ENREF_98)]; also involved in the production of ROS |
| 2 | α-Adrenergic signalling | 0.000791 | 0.019 | Stress response; glycogenolysis; elevation of intracellular Ca^2+^ [[68](#_ENREF_68)] |
| 3 | G beta gamma signalling | 0.000828 | 0.017 | Control calcium signalling and protein kinase A signalling pathways in its down-stream effects [[99](#_ENREF_99), [100](#_ENREF_100)] |
| 4 | Corticotropin releasing hormone signalling | 0.00124 | 0.015 | Stress response and secretion of glucocorticoids; cell death; activates PKC-dependent and calcium-activated pathways respectively [[101-103](#_ENREF_101)] |
| 5 | P2Y purigenic receptor signalling | 0.00140 | 0.015 | Angiogenesis; wound healing; cellular proliferation; ion transport [[104-106](#_ENREF_104)] |
| 6 | Protein kinase A signalling | 0.0094 | 0.006 | Raised level of intracellular Ca2+; proteasomal degradation; Glycolysis; Lipolysis [[107](#_ENREF_107), [108](#_ENREF_108)] |
| 7 | FcγRIIB signalling in B lymphocytes | 0.0202 | 0.017 | Hypersensitivity response; inflammatory response [[109](#_ENREF_109), [110](#_ENREF_110)] |
| 8 | CCR5 signalling in macrophages | 0.0383 | 0.011 | Inflammatory response; cytokine signalling; cellular immune response [[44](#_ENREF_44), [45](#_ENREF_45), [111](#_ENREF_111)] |
| 9 | Chemokine signalling | 0.0343 | 0.014 | Inflammatory response; intracellular calcium release; production of reactive oxygen species (ROS) and changes in actin polymerization [[44](#_ENREF_44), [45](#_ENREF_45), [112](#_ENREF_112), [113](#_ENREF_113)] |
| 10 | Phospholipid degradation | 0.0367 | 0.011 | Production of PLA2; phospholipid degradation [[114](#_ENREF_114), [115](#_ENREF_115)] |

*^1^P*-value: Shows the strength of the association between a specific pathway and the uploaded dataset. Small *P* values suggest that the probability of a spurious association between the data and this pathway is unlikely (<http://ingenuity.force.com/ipa/>).

^2^Ratio: The number of genes in a given pathway divided by the total number of genes that make up that pathway (<http://ingenuity.force.com/ipa/>).

Table S6 Pathways selected from category V cluster analysis

| **No.** | **Pathway title** | ***P*-value^1^** | **Ratio^2^** | **Functions and diseases** |
| --- | --- | --- | --- | --- |
| 1 | MIF-mediated glucocorticoid regulation | 0.015 | 0.024 | Counter-regulation of the anti-inflammatory effects of glucocorticoids; cellular immune response; organismal injuries and abnormalities [[116-118](#_ENREF_116)] |
| 2 | MIF regulation of innate immunity | 0.020 | 0.020 | Proliferation and trafficking of immune cells; cellular immune response [[119](#_ENREF_119), [120](#_ENREF_120)] |
| 3 | Eicosanoid signalling | 0.024 | 0.013 | Inflammation; vascular permeability, and allergic reaction [[121](#_ENREF_121), [122](#_ENREF_122)] |
| 4 | Role of MAPK signalling in the pathogenesis of influenza | 0.033 | 0.015 | Oxidative stress and apoptosis through production of prostaglandin E2 [[71](#_ENREF_71)] |
| 5 | Phospholipid degradation | 0.039 | 0.011 | PLA2, phospholipid degradation [[114](#_ENREF_114), [115](#_ENREF_115)] |

^1^*P*-value: Shows the strength of the association between a specific pathway and the uploaded dataset. Small *P* values suggest that the probability of a spurious association between the data and this pathway is unlikely (<http://ingenuity.force.com/ipa/>).

^2^Ratio: The number of genes in a given pathway divided by the total number of genes that make up that pathway (http://ingenuity.force.com/ipa/).

Table S7 Pathways selected from category VI cluster analysis

| **No.** | **Pathway title** | ***P*-value** | **Ratio** | **Functions and diseases** |
| --- | --- | --- | --- | --- |
| 1 | Role of JAK1, JAK2 and TYK2 in interferon signalling | 0.0221 | 0.037 | Cytokine signalling; haematological system development and function; haematopoiesis [[123](#_ENREF_123), [124](#_ENREF_124)] |
| 2 | Interferon signalling | 0.0312 | 0.028 | Inflammatory response; cellular immune response; cytokine signalling; organismal injury and abnormalities [[58](#_ENREF_58), [125](#_ENREF_125)] |
| 3 | TNFR1 signalling | 0.0438 | 0.019 | Apoptosis; cytokine signalling [[126](#_ENREF_126)] |

#### ^1^*P*-value: Shows the strength of the association between a specific pathway and the uploaded dataset. Small *P* values suggest that the probability of a spurious association between the data and this pathway is unlikely (<http://ingenuity.force.com/ipa/>).

#### ^2^Ratio: The number of genes in a given pathway divided by the total number of genes that make up that pathway (<http://ingenuity.force.com/ipa/>).

Table S8 Selected pathways from IPA analysis of BioLayout filtered genes

| **No.** | **Pathway titles** | ***P*-value^1^** | **Ratio^2^** | **Functions and diseases** |
| --- | --- | --- | --- | --- |
| 1 | Granzyme B signalling | 0.00021 | 0.312 | Oxidative stress; Cell death; Cellular immune response [[127](#_ENREF_127), [128](#_ENREF_128)] |
| 2 | Toll-like receptor | 0.00225 | 0.127 | Apoptosis [[129](#_ENREF_129), [130](#_ENREF_130)] |
| 3 | Type I diabetes Mellitus signalling | 2.35E00 | 8.26E-02 | Tissue damage and muscle weakness [[131](#_ENREF_131)] |
| 4 | Cytotoxic T lymphocyte mediated apoptosis of target cells | 0.00457 | 0.058 | Cell death [[127](#_ENREF_127), [132](#_ENREF_132)] |
| 5 | Apoptosis signalling | 0.00746 | 0.094 | Apoptosis [[133](#_ENREF_133), [134](#_ENREF_134)] |
| 6 | Role of MAPK signalling in the pathogenesis of influenza | 0.00911 | 0.106 | Oxidative stress; apoptosis [[71](#_ENREF_71)] |
| 7 | Lymphotoxin β receptor signalling | 0.0175 | 0.098 | Cell death [[135](#_ENREF_135), [136](#_ENREF_136)] |
| 8 | IL-1 signalling |  | 0.075 | Inflammation [[137-139](#_ENREF_137)] |

^1^*P-value*: Shows the strength of the association between a specific pathway and the uploaded dataset. Small P values suggest that the probability of a spurious association between the data and this pathway is unlikely (<http://ingenuity.force.com/ipa/>).

^2^Ratio: The number of genes in a given pathway divided by total number of genes that make up that pathway (<http://ingenuity.force.com/ipa/>)
